# Supplementary material for: Empirical methods for controlling false positives and estimating confidence in ChIP-Seq peaks
Source: BMC Bioinformatics. 2008 Dec 5;9:523. doi: 10.1186/1471-2105-9-523 (PMC2628906; doi:10.1186/1471-2105-9-523)
Supplement: Additional File 1 — A variety of html documents from the USeq web site detailing the available applications, their best usage, output file type descriptions, command line menus, etc. [file 1471-2105-9-523-S1.zip › USeqUserGuides/JavaDoc.zip!/JavaDoc/util/bio/digest/NEBParser.html]

NEBParser


|  |  |  |
| --- | --- | --- |
| **Overview** \| **Package** \| **Class** \| **Use** \| **Tree** \| **Deprecated** \| **Index** \| **Help** | |  |
| **PREV CLASS**   **NEXT CLASS** | **FRAMES**    **NO FRAMES**     **All Classes** |  |
| SUMMARY: NESTED \| FIELD \| CONSTR \| METHOD | DETAIL: FIELD \| CONSTR \| METHOD |  |


---


## util.bio.digest Class NEBParser

```
java.lang.Object
  util.bio.digest.NEBParser
```

---

``` public class NEBParser extends java.lang.Object ```

Parses a New England Biolabs restriction enzyme file into an array of `Enzyme`s.

---

**Constructor Summary**

`NEBParser()`


| **Method Summary** | |
| --- | --- |
| `Enzyme[]` | `getEnzymes()` |
| `java.lang.String` | `getHeader()` |
| `Enzyme[]` | `makeEnzymeArray(java.lang.String gtype2c, int minLengthRecogSeq)` |

**Methods inherited from class java.lang.Object**

`equals, getClass, hashCode, notify, notifyAll, toString, wait, wait, wait`

**Constructor Detail**

### NEBParser

```
public NEBParser()
```


**Method Detail**

### getHeader

```
public java.lang.String getHeader()
```

---


### makeEnzymeArray

```
public Enzyme[] makeEnzymeArray(java.lang.String gtype2c,
                                int minLengthRecogSeq)
```

---


### getEnzymes

```
public Enzyme[] getEnzymes()
```


---


|  |  |  |
| --- | --- | --- |
| **Overview** \| **Package** \| **Class** \| **Use** \| **Tree** \| **Deprecated** \| **Index** \| **Help** | |  |
| **PREV CLASS**   **NEXT CLASS** | **FRAMES**    **NO FRAMES**     **All Classes** |  |
| SUMMARY: NESTED \| FIELD \| CONSTR \| METHOD | DETAIL: FIELD \| CONSTR \| METHOD |  |


---
